# Novel Chemical Ligands to Ebola Virus and Marburg Virus Nucleoproteins Identified by Combining Affinity Mass Spectrometry and Metabolomics Approaches

**DOI:** 10.1038/srep29680

**Published:** 2016-07-12

**Authors:** Xu Fu, Zhihua Wang, Lixin Li, Shishang Dong, Zhucui Li, Zhenzuo Jiang, Yuefei Wang, Wenqing Shui

**Affiliations:** 1 College of Biotechnology, Tianjin University of Science & Technology, Tianjin 300457, China; 2 High-throughput Molecular Drug Discovery Center, Tianjin Joint Academy of Biotechnology and Medicine, Tianjin 300457, China; 3 College of Pharmacy, Nankai University, Tianjin 300071, China; 4 Tianjin Institute of Industrial Biotechnology, Chinese Academy of Sciences, Tianjin 300308, China; 5 Tianjin State Key Laboratory of Modern Chinese Medicine, Tianjin University of Traditional Chinese Medicine, Tianjin 300193, China; 6 iHuman Institute, ShanghaiTech University, Shanghai 201210, China

## Abstract

The nucleoprotein (NP) of Ebola virus (EBOV) and Marburg virus (MARV) is an essential component of the viral ribonucleoprotein complex and significantly impacts replication and transcription of the viral RNA genome. Although NP is regarded as a promising antiviral druggable target, no chemical ligands have been reported to interact with EBOV NP or MARV NP. We identified two compounds from a traditional Chinese medicine Gancao (licorice root) that can bind both NPs by combining affinity mass spectrometry and metabolomics approaches. These two ligands, 18β-glycyrrhetinic acid and licochalcone A, were verified by defined compound mixture screens and further characterized with individual ligand binding assays. Accompanying biophysical analyses demonstrate that binding of 18β-glycyrrhetinic acid to EBOV NP significantly reduces protein thermal stability, induces formation of large NP oligomers, and disrupts the critical association of viral ssRNA with NP complexes whereas the compound showed no such activity on MARV NP. Our study has revealed the substantial potential of new analytical techniques in ligand discovery from natural herb resources. In addition, identification of a chemical ligand that influences the oligomeric state and RNA-binding function of EBOV NP sheds new light on antiviral drug development.

Ebola viruses (EBOV) and Marburg viruses (MARV) that cause severe hemorrhagic fever with case fatality rates approaching 90% during some outbreaks are undoubtedly significant threats to global human health^1^,^2^. The recent largest filovirus epidemic in Western Africa caused by EBOV Makona variant, and the import of EBOV to non-African countries highlights the public health impact of these pathogens.

EBOV has a single-stranded RNA (ssRNA) genome that is transcribed upon virus entry into the host cytosol before generation of at least eight distinct viral proteins^3^,^4^,^5^,^6^. Viral genome replication and mRNA transcription are carried out by the viral RNA-dependent RNA polymerase complex (RDRP)^4^. This complex consists of the viral polymerase (L), the viral nucleoprotein (NP), along with polymerase cofactors VP35 and VP30. For all members in the filovirus family, the single-stranded viral genomic RNAs are encapsidated by NPs so that the viral genome can be protected from recognition by the cellular innate immune system or degradation by ribonucleases^7^,^8^.

The prominent role of virus NPs as a valid target in antiviral drug development has been manifested in studies on influenza A virus (IAV) NP. Two compounds, nucleozin and RK424, that are found to target IAV NP and alter its oligomeric state showed potent antiviral activity against IAV infection *in vitro* and *in vivo*^9^,^10^. However, no chemical ligands have been reported towards EBOV NP or MARV NP. The only known ligand of EBOV NP is a peptide derived from the protein component VP35 within the viral nucleocapsid^11^,^12^. This VP35 peptide binds NP with high affinity and specificity, inhibits NP oligomerization, and releases RNA from NP-RNA complexes *in vitro*. Previous elegant structural work on the VP35 peptide bound EBOV NP complex reveals how the peptide ligand occludes a large surface area important for NP assembly, NP-RNA interactions, and viral RNA synthesis^11^,^12^. These results strongly imply that EBOV NP can be also a good target for therapeutic development.

Affinity mass spectrometry (MS) has emerged as a versatile and complementary approach for ligand screens against drug targets and played an increasingly vital role in early drug discovery^13^,^14^,^15^,^16^. An important advantage of MS-based ligand discovery over other biophysical techniques for biomolecualr interaction analysis lies in its ability to detect specific ligands from a complex compound mixture such as combinatorial libraries^17^,^18^,^19^. We have established affinity MS-based workflows for identification and affinity measurement of chemical ligands towards several antiviral protein targets^19^,^20^,^21^. In comparison to synthetic compound mixture screens, the potential of affinity MS has not been as widely explored in ligand screens from natural product extracts due to the structural complexity and abundance variation of different constituents.

In this work, we combined affinity MS and metabolomic approaches for identification of chemical ligands to EBOV NP and MARV NP from the crude extract of a renowned traditional Chinese medicine (TCM) Gancao (licorice root). Gancao is the dried root of *Glycyrrhiza uralensis* Fisch (licorice), *G. inflata* Bat., or *G. glabra* L. It has shown a wide variety of therapeutic properties such as antiviral, anti-hepatotoxic, anti-inflammatory, anti-leukemogenic, and anti-carcinogenic effects in clinical treatment of liver diseases^22^,^23^,^24^,^25^,^26^. Here, two compounds were discovered from Chinese licorice to bind both EBOV NP and MARV NP. They were verified by defined compound mixture screens and further characterized with individual ligand binding assays. Accompanying biophysical analyses demonstrate that binding of one ligand to EBOV NP significantly reduces protein thermal stability, induces formation of large NP oligomers and disrupts he critical association of viral ssRNA with NP complexes. Our study has revealed the substantial potential of affinity MS coupled to metabolomic techniques in ligand discovery from natural herb resources for the development of small-molecule therapies.

## Results

### Screening for chemical ligands to Ebola virus and Marburg virus NPs from Chinese licorice extracts

**Figure 1:**
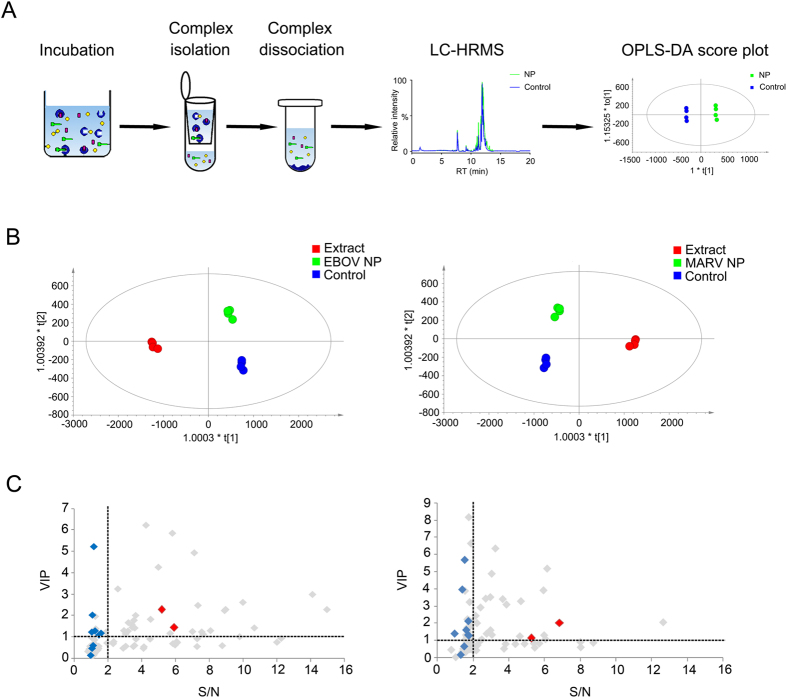
Identification of chemical ligands bound to Ebola virus and Marburg virus NPs from Chinese licorice. (**A**) The workflow of combining affinity MS and metabolomics approaches for ligand discovery towards NPs. (**B**) OPLS-DA score plots of the crude extracts, the NP incubation samples and the control samples show clear separation of three groups in the data sets of EBOV NP (left) and MARV NP (right). (**C**) VIP and S/N plots of features detected in the protein incubation sample and control from the EBOV NP experiment (left) and MARV NP experiment (right). Each grey symbol represents a feature detected in four independent replicates (RSD of S/N ratios across replicates < 30%). Each feature also matches its accurate mass with the Chinese licorice compound database. Red symbols annotate two putative ligands bound to NPs (GC7 and GC13). Blue symbols annotate the rest of 11 constituents in Chinese licorice that do not bind NPs. All color-coded compounds are identified by HRMS and MSMS analysis according to reference standards.

To comprehensively screen the crude extract of Chinese licorice for virus NP ligand discovery, we devised a workflow combining affinity mass spectrometry and metabolomics approaches (Fig. 1A). The recombinant core domains of Ebola virus NP (EBOV NP, amino acids 36–351) and Marburg virus NP (MARV NP, amino acids 19–370) were purified and separately incubated with Chinese licorice extract before ultrafiltration was performed to isolate ligand-bound NP complexes. The compound mixture dissociated from the NP complexes or the protein-free control was subjected to metabolomic analysis using liquid chromatography coupled to high-resolution mass spectrometry (LC-HRMS) (full datasets in Supplemental Table S1). A multivariate model based on orthogonal partial least-squares discriminant analysis (OPLS-DA) was then created to differentiate the compound profiles from the crude extracts, the protein incubation samples and the control samples. In both types of NP experiments, there was distinct separation of three sample groups, indicating that the composition of compounds associated with the NP complex was substantially deviated from that in the crude extract and the control (Fig. 1B). We then applied multiple criteria to select putative ligands specifically enriched in the EBOV NP or MARV NP complexes which include: 1) variable influence on projection (VIP) score > 1.0, representing compounds most implicated in the difference between the NP complex and control^27^,^28^; 2) average S/N value > 2.0 and RSD < 30%, indicating significant change of peak intensity of the compound in the protein complex relative to control^19^,^20^; 3) matching the accurate mass of a putative ligand to the TCM compound database for Chinese licorice (see details in Experimental). Using this set of stringent criteria, we identified 30 and 22 components from the licorice extract to be candidate ligands bound to EBOV NP or MARV NP, respectively (Fig. 1C, Table S1).

### Structural elucidation of viral NP ligands

Although putative structures can be assigned to compounds of interest based on HRMS and MS/MS analysis^29^, unambiguous structural elucidation requires strict confirmation with reference standards. To this end, we obtained 14 pure standards (GC1-GC14) known to be licorice constituents through commercial sources or purification from licorice extract^30^ (compound list in Supplemental Figure S1). Identification of these compounds in the NP incubation sample and the control was based on matching both retention time (<0.1 min shift) and accurate mass (<0.01 Da deviation) to the reference. By this means, eleven features corresponding to 13 licorice constituents (containing two pairs of isomers) were identified. They were color coded in the VIP and S/N plots that include all reproducibly detected features matched with the Chinese licorice compound database (Fig. 1C).

**Figure 2:**
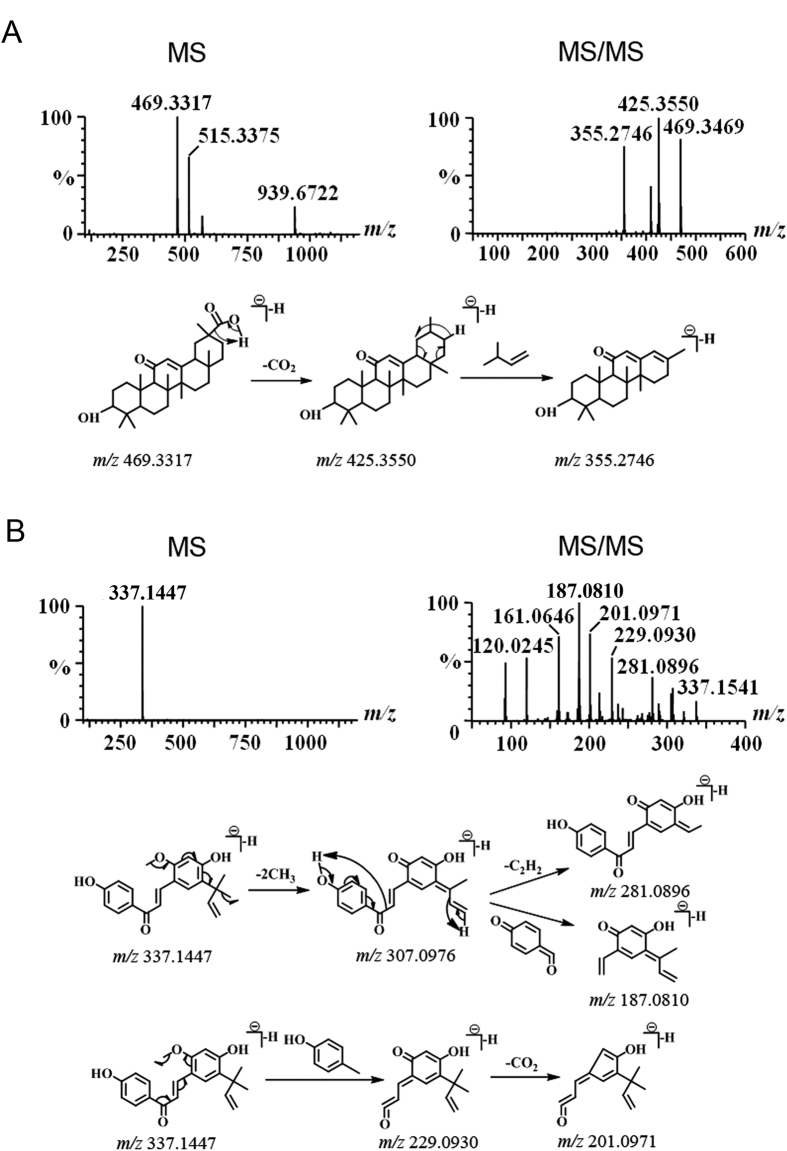
Structural elucidation of GC7 (A) and GC13 (B) by MS and MSMS analysis. The proposed fragmentation pathway is shown below mass spectra.

Among the identified licorice constituents, we found GC7/10 (glycyrrhetinic acid) and GC13 (licochalcone A) met the aforementioned thresholds for both EBOV NP and MARV NP ligands (red symbols in Fig. 1C). It is noteworthy that GC7 (18β-glycyrrhetinic acid) and GC10 (18α-glycyrrhetinic acid) are a pair of diastereo-isomers differing only in their C_18_–H–, *trans*-, and *cis*-configuration, and they coeluted under the LC condition of this study. High-resolution MS and MSMS spectra were acquired on GC7/10 and GC13 in the protein incubation sample to support structural elucidation (Fig. 2). In addition, NMR analysis of the corresponding LC fractions for the putative ligands revealed that 18β-glycyrrhetinic acid was the dominant isomer in the licorice extract (Supplemental Figure S2).

### Validation of ligand binding to NP using pure standards

**Figure 3:**
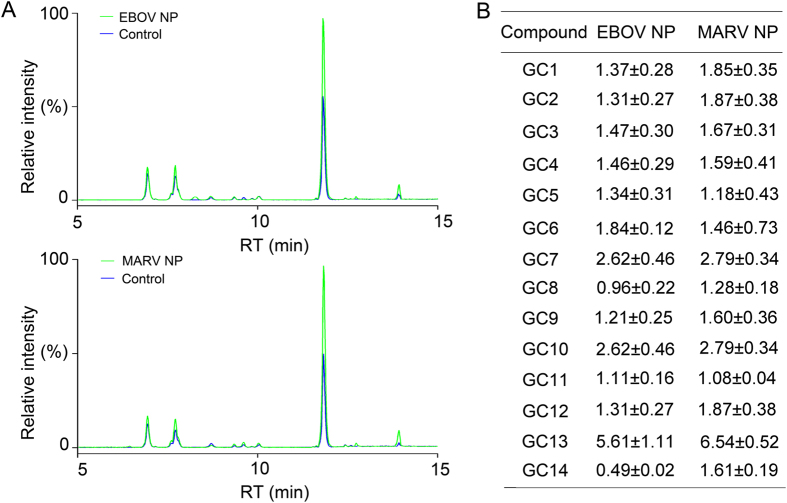
Validation of NP ligands in compound mixture screens. (**A**) LC-HRMS chromatograms of the protein incubation sample and the control from the EBOV NP experiment (upper) and MARV NP experiment (lower). The NP protein was incubated with a mixture of 14 pure compounds known to be licorice constituents and then ligands were identified by affinity MS analysis. (**B**) S/N ratios of specific compounds from the mixture screen experiment. Note that GC2/GC12 and GC7/GC10 show identical S/N ratios as they are isomeric pairs that cannot be distinguished under the LC condition. GC7/GC10 and GC13 with S/N > 2 are considered ligands to both NPs.

To verify binding of ligands discovered from the licorice extract and evaluate other known licorice constituents, we incubated the EBOV NP or MARV NP protein with a mixture of fourteen pure standards at an equal amount for affinity MS analysis. Figure 3A shows the LC-HRMS chromatograms for the protein incubation sample and the control in two types of NP experiments. Compounds with an average S/N ratio > 2 (RSD < 30%) indicate significant increase of abundance in the protein complex and thus are considered ligands to the NP target. Both GC7/10 and GC13 from the compound mixture were highly enriched in EBOV NP and MARV NP complexes whereas none of the other licorice constituents showed significant binding (Fig. 3B).

To further characterize ligand binding properties, we incubated the NP protein with GC7, GC10 and GC13 separately prior to affinity MS analysis. Using previously developed methods^21^, the S/N ratio, binding fraction and equilibrium dissociation constant (K_d_) were obtained for individual compounds (Table 1). Interestingly, for the two diostereo-isomers, it is GC7 not GC10 that is the positive binder of EBOV NP and MARV NP with medium μM affinity. GC13 was also verified to be a ligand of both NPs yet with very weak affinity (>1 mM) (Table 1). In addition to affinity MS assays, we performed conventional SPR experiments to verify binding of GC7 and GC13 to both NP targets. In accordance with our affinity MS results, GC10 and several other tested licorice components also showed negative SPR responses (Supplemental Table S2).

Taken together, binding assays on the compound mixture and pure compounds by affinity MS and SPR concordantly validated that GC7 and GC13 are ligands to EBOV NP and MARV NP. Consistent findings between the crude extract analysis and following validation assays suggest that our integrated affinity MS and metabolomic workflow is highly effective in discovery of new ligands from complex TCM extracts.

### GC7 binding reduces EBOV NP thermal stability and promotes NP oligomerization

The microscale fluorescent thermal shift assay was conducted to assess NP thermal stability upon different ligand binding (Table 2). Strikingly, GC7 largely reduced EBOV NP thermal stability as it caused 9 °C decrease of the protein melting temperature. To the best of our knowledge, it is the first report of a compound that induces such substantial instability of a viral NP, which is strongly suggestive of considerable conformational changes of EBOV NP upon GC7 binding. GC13 marginally affected EBOV NP thermal stability and no other tested licorice constituents had such effect. By contrast, all tested compounds including GC7 and GC13 had little influence on MARV NP thermal stability (Table 3), suggesting the specificity of GC7 in impairing EBOV NP stability.

The oligomeric state of viral NPs is closely related to their functions in replication and transcription of viral RNA genomes^4^,^10^,^11^,^12^. The N-terminal truncation construct of EBOV NP (residues 36–351) used in our study entails the folded core domain which stays as an RNA-free, monomeric form in solution^31^. However, addition of GC7 to the EBOV NP solution shifted the monomer to much larger molecular weight species as shown by size-exclusion chromatography with in-line multiangle light scattering (SEC-MALS) (Table 3). Increased molar excess of GC7 eventually produced super-large complexes of EBOV NP. This ligand-bound EBOV NP core region gave rise to a monodispersed peak eluting in the void volume of SEC that closely resembled the peak of another construct 1–450 well characterized to form large homo-oligomeric complexes^11^,^12^ (Supplemental Figure S3). These results indicated GC7 shifted the equilibrium of EBOV NP towards the native oligomeric state of NPs. Notably, addition of the structural enantiomer GC10 converted a small portion of the monomeric EBOV NP to oligomers. In contrast, GC13 and another licorice constituent GC2 had very little effect in mediating oligomerization of EBOV NP (Table 3). The MARV NP truncation construct (residues 19–370) used in our study formed a mixed population of monomer, tetramer and large oligomer in solution. Interestingly, binding of GC7 to our MARV NP disrupted its tetramer conformation and increased the fraction of monomers in a dose-dependent way (Table 3). On the contrary, incubation of MARV NP with GC13 largely promoted tetramer formation (>2-fold increase) whereas reducing the fraction of monomers. GC10 and GC2 did not induce significant changes in the monomer and oligomer distribution of MARV NP (Table 3).

Taken together, among the compounds investigated in our study, GC7 displayed the most profound impact on both thermal stability and oligomerization of EBOV NP, encouraging us to further characterize its binding mode and *in vitro* bio-activity.

### New ligands target the RNA-binding groove of Ebola virus NP

**Figure 4:**
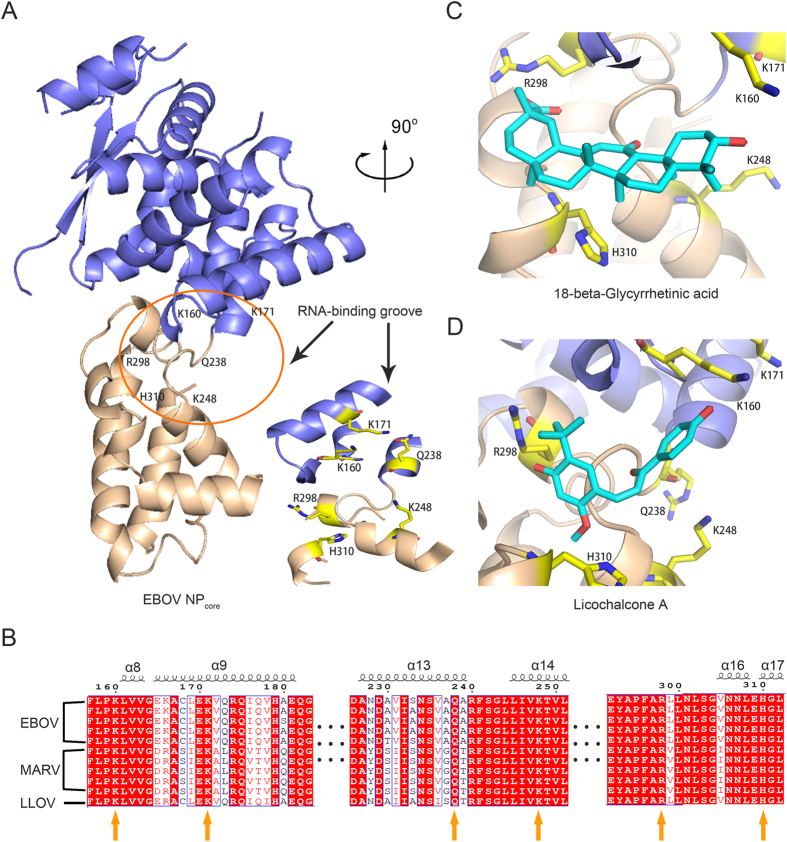
Potential interaction of new ligands with the RNA-binding groove of Ebola virus NP. (**A**) The crystal structure of EBOV NP_core_ containing the N-lobe (in blue) and C-lobe (in brown). The RNA-binding pocket surrounded by several residues is marked by a circle and enlarged in the side stereoview. (**B**) Primary sequence alignment of members of the *filoviridae* family. Residues in the RNA-binding groove are indicated by yellow arrows. Docking model of GC7 (**C**) and GC13 (**D**) interacting with the RNA-binding groove of EBOV NP.

The crystal structure of EBOV NP (PDB code 4Z9P) solved in a previous study is featured by two identifiable lobes, an N-lobe spanning NP residues 36 to 240 and a C-lobe from 241 to 351 that are connected together by a flexible hinge located within the C-lobe^31^. From this structure, a highly positively charged groove located at the interface between the N- and C-lobes constitutes a potential RNA-binding pocket (Fig. 4A). In this pocket, the major positive-charged crevice involves residues K160, K171, R174, and K248 and the adjacent minor positive-charged region consists of residues R205, K211, and R298^31^. These key basic residues are highly conserved across not only the Ebola virus genus but the whole filovirus family as well (Fig. 4B). They are reported to play essential roles in viral RNA encapsidation and EBOV proliferation in host cells^11^,^32^,^33^,^34^.

**Figure 5:**
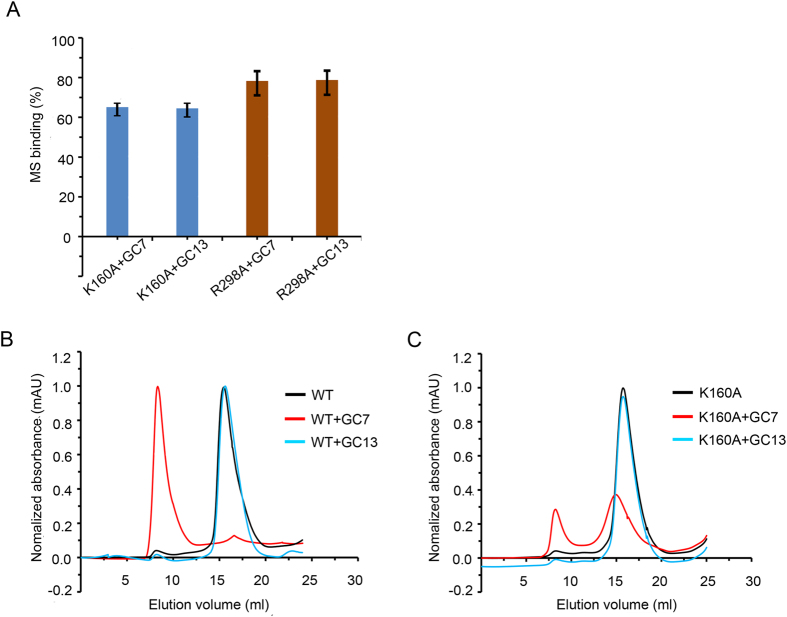
K160 is engaged in ligand binding and shift of NP oligomeric state. (**A**) Binding efficiency of GC7 or GC13 to a specific EBOV NP mutant normalized to ligand binding to wild-type NP (defined as 100%) measured by affinity MS. Error bars represent SD from independent experiments in triplicate. (**B**) SEC chromatograms of wild-type EBOV NP alone or incubated with a specific ligand. (**C**) SEC chromatograms of EBOV NP K160A mutant alone or incubated with a specific ligand.

Based on the apo protein structure, we employed *in silico* docking analysis to create a potential model for binding of GC7 and GC13 to EBOV NP. The predicted ligand binding site involves residues K160 and R298 that possibly make hydrogen bonds with both ligands (Fig. 4C,D). We prepared EBOV NP mutants harboring alanine substitution at K160 and R298 to examine ligand binding capacity using our affinity MS approach. Compared to the wild-type protein, mutation at K160 resulted in nearly 40% loss of binding of both GC7 and GC13, and mutation at R298 reduced binding efficiency of two ligands to a less extent (Fig. 5A). Importantly, the predicted binding free energy for GC7 (−5.88 kcal/mol) is much lower than that for GC10 (−2.75 kcal/mol), which provides the molecular basis for selective binding of one diostereo-isomer GC7 to the protein target. Furthermore, whereas the binding of GC7 strongly induced oligomerization of the wild-type EBOV NP, less obvious shift of the oligomeric state with the same ligand was observed for mutant K160A (Fig. 5B,C).

These results suggest that two new ligands discovered in our study interact with the RNA-binding groove of EBOV NP through specific residues predicted by the docking model, and K160 is further engaged in GC7-triggered NP oligomerization.

### GC7 disrupts EBOV NP-RNA interaction

**Figure 6:**
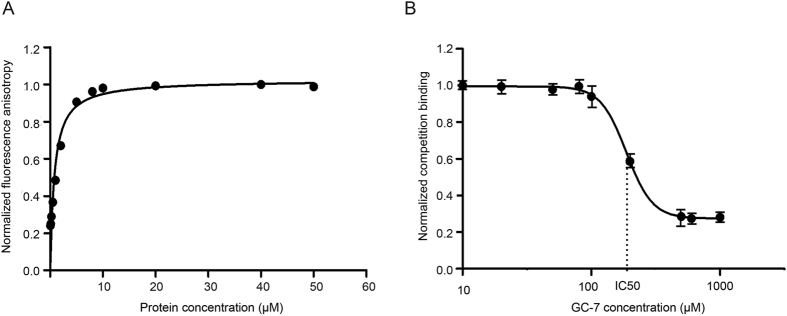
GC7 disrupts ssRNA association with oligomeric EBOV NP complexes. (**A**) Measurement of ssRNA binding to oligomeric EBOV NP 36–450 at increasing concentrations by fluorescence anisotropy. (**B**) Competitive binding between GC7 and fluorophore-labeled ssRNA to oligomeric EBOV NP 36–450. IC_50_ is derived at the concentration of GC7 that inhibits ssRNA binding to NP oligomers by 50%. Error bars represent SD from independent experiments in triplicate.

To evaluate the effect of the ligand GC7 in mediating EBOV NP interaction with viral ssRNA, we generated another construct (residue 36–450) in order to purify RNA-free oligomerized EBOV NP. The bacterial RNA co-purified with the protein was removed by collecting the monomer fraction eluted from SEC using high-salt buffer (1.5 M NaCl) (Supplemental Figure S4A). Subsequent dilution to the low-salt condition (200 mM NaCl) triggered spontaneous NP oligomerization confirmed by SEC analysis (Supplemental Figure S4B). The resulting oligomeric RNA-free EBOV NP 36–450 readily binds to an 18 nucleotide ssRNA with low micromolar affinity (~5 μM) measured by fluorescence anisotropy (Fig. 6A). Next a competitive binding assay was conducted by first incubating the monomer of EBOV NP 36–450 with GC7 at various concentrations before diluting the mixture and adding the RNA probe. GC7 incubation with the NP monomer caused protein oligomerization. Importantly, this ligand when bound to the NP oligomer was found to compete with ssRNA for interaction with NP complexes. Increasing GC7 concentration during incubation resulted in a proportional loss of ssRNA from the RNA-NP complex, yielding a half maximal inhibitory concentration (IC_50_) of ~160 μM (Fig. 6B). These results indicate that the new ligand GC7 disrupts the interaction of EBOV NP with viral ssRNA through either directly occluding the RNA-binding cleft or inducing a conformational change of NP oligomers that would impair ssRNA association.

## Discussion

Here, our study reports two novel chemical ligands targeting Ebola virus and Marburg virus NPs. They were identified from Chinese licorice by combining affinity MS and metabolomic techniques. Traditionally, ultrafiltration-HPLC was employed for ligand discovery from natural product or medicinal plant extracts^15^,^29^,^35^,^36^. Most previous studies used HPLC coupled to a UV/vis detector to search for compounds that are enriched in the protein incubation with natural product extracts, and then applied mass spectrometry analysis to structural identification of these potential ligands. However, limited sensitivity of the UV/vis detector substantially lowers the chance of capturing medium to low abundant species in the complex crude extracts. In comparison to dozens of peaks typically observed in the ultrafiltration-HPLC chromatograms^29^,^35^,^36^, the HRMS-based metabolomic workflow enables detection of over 1500 features in our NP incubation sample with the licorice extract. Quantitative comparison and OPLS-DA analysis of these features allowed us to evaluate binding capability of the majority of constituents in Chinese licorice. At the end, 30 and 22 components were identified to be putative ligands bound to EBOV NP or MARV NP using this new workflow. Simultaneous MSMS analysis in the metabolomic experiment facilitates structural identification of putative ligands. Using the available reference compounds, structures of two ligands were unambiguously determined.

GC7 (18β-glycyrrhetinic acid) and GC13 (licochalcone A) represent the first chemical ligands reported to partake in interaction with filovirus NPs. Accompanying biophysical analyses demonstrate that GC7 binding impairs the thermal stability of EBOV NP core domain, triggers protein oligomerization, and disrupts viral RNA association with oligomeric NP complexes. Prior to our study the only documented ligand to EBOV NP is a peptide derived from EBOV VP35 which is a protein component of the viral RNA-dependent RNA polymerase complex (RDRP)^11^,^12^. Notably, this VP35 N-terminal peptide can maintain NP in a non-oligomeric and RNA-free state (NP^0^), yet it cannot support viral RNA synthesis *in trans*. Thus the previous work on VP35 peptide chaperoning the NP assembly granted a model where Ebola VP35 peptide binding to NP prevents NP from associating with noncognate RNA (*i.e.*, cellular and non-genomic viral RNA)^12^. Furthermore, VP35 is speculated to facilitate transfer of the monomeric and template-free NP to the viral template RNA to initiate RNA synthesis^11^. By contrast, the chemical ligand GC7 discovered in our study exerts an effect opposite to the VP35 peptide which is to induce formation of large oligomeric complexes of the NP core domain (Fig. 5B, Table 3). This result reminded us of nucleozin, a chemical compound targeting influenza A NP that can induce the formation of NP aggregates and antagonize its nuclear accumulation, leading to cessation of viral replication *in vitro*^9^. Another chemical RK424 which disrupts viral RNA-induced oligomerization of influenza A NP, also showed potent antiviral activity against many different subtypes of influenza A virus *in vitro* and *in vivo*^10^. However, no compounds capable of influencing the NP oligomeric state have been discovered towards Ebola virus up to date. As the NP protein is an integral part of the viral nucleocapsid and is intimately associated with the viral RNA template, its oligomeric state is particularly important for maintaining template integrity and providing template access to the viral RDRP^11^. The ability of GC7 to disrupt ssRNA association with NP complexes further indicates this ligand upon binding to the RNA-free NP monomer may induce a significant conformational change of the resulting oligomers that they no longer interact with viral ssRNA as effectively as the native NP oligomers. Therefore, whether GC7 and its derivatives have any pharmacological activity on Ebola virus life cycle would be a very intriguing question to address in the future.

According to our *in silico* docking results, both GC7 and GC13 are predicted to interact with the RNA-binding groove of EBOV NP which is composed of certain basic residues (K160, K171, K248 and R298). In particular, K160 and R298 possibly make hydrogen bonds with the hydroxyl groups of two ligands (Fig. 4C,D). Substitution of K160 with alanine resulted in significant loss of binding capacity of GC7, and this residue is further engaged in GC7-induced NP oligomerization (Fig. 5). We also noticed that substitution of either K160 or R298 did not completely abrogate binding capacity of GC7 and GC13, suggesting there may be other residues engaged in ligand interactions. Importantly, these residues comprising the GC7/GC13-binding site are well conserved across the whole filovirus family (Fig. 4B) and known to play essential roles in encapsidation of the viral genome and EBOV proliferation^31^,^38^,^39^,^40^, which sheds light on antiviral drug development. Considering the strong homology of the core domain between EBOV NP and MARV NP, we would speculate GC7 and GC13 might interact with the RNA-binding groove of MARV NP in a similar fashion. It is noteworthy that GC7 exhibits much less influence on the thermal stability and oligomerization state of MARV NP compared to EBOV NP, probably because the protein was purified in a mixed form of monomer, tetramer and oligomer (Table 3). In addition, lack of protein structural data for MARV NP renders it more difficult to predict ligand binding mode. Therefore, further structural and biochemical study of the NP-ligand complex is needed to definitively identify the precise interaction mode.

GC7 (18β-glycyrrhetinic acid) is in fact the bio-transformed metabolite of glycyrrhizin by intestinal flora in the human body. Glycyrrhizin is a well-characterized major component of Chinese licorice and possesses a wide range of pharmacological and biological activities^24^. Glycyrrhizin in line with glycyrrhetinic acid have been developed in China and Japan as an anti-inflammatory, antiviral, and antiallergic drug for liver disease treatment^24^. It is of our particular notion that glycyrrhizin was reported to inhibit the replication of SARS-associated coranovirus and Hepatitis C virus^37^,^38^,^39^. Furthermore, glycyrrhizin exerts antiviral effects in H5N1 influenza A virus-infected cells by inhibiting virus replication and pro-inflammatory gene expression^40^. Interestingly, although glycyrrhizin (*i.e.* GC6) is an abundant constituent of Chinese licorice, we did not observe its direct binding to EBOV NP or MARV NP in the crude extract or the compound mixture screens (Figs 1 and 3), nor did it show any influence on NP thermal stability (Table 2). Our results clearly indicate that it is GC7, the primary metabolite of glycyrrhizin, that targets viral NPs directly and exhibits multiple biological activities against EBOV NP. These findings would shed new light on our understanding of the antiviral mechanism of Chinese licorice. Given the structural diversity and novelty of numerous compounds in natural products and traditional Chinese medicine, our study presents a promising new approach to mine the great resource of natural herbs for ligand discovery towards therapeutic targets and thus aid potential drug development.

## Methods

### Materials and Reagents

Ammonium acetate, formic acid, Tris and NaCl were purchased from Sigma–Aldrich (St. Louis, MO, USA). Peptone, yeast powder, IPTG, ampicillin and imidazole were purchased from Sangon Biotech Shanghai Co., Ltd (Shanghai, China). Methyl alcohol and acetonitrile of HPLC-grade were purchased from Merck (Darmstadt, Germany), Ultra-pure water was purified with the Milli-Q system (Millipore, USA). Centrifugal ultrafiltration units (30 kDa) were purchased from Sartorius (Germany). Protein Thermal Shift Dye Kit^TM^ was purchased from Thermo Fisher Scientific (Massachusetts, USA). Fourteen standards of known licorice constituents listed in Supplemental Figure S1 were purchased from Chengdu Herbpurify Co., Ltd (Chengdu, China) and National Institute for Food and Drug Control (Beijing, China), or isolated using a reported protocol^30^ by the modern Traditional Chinese Medicine key laboratory at Tianjin University of Traditional Chinese Medicine, China.

### Protein expression and purification

Cloning and purification was performed as described previously^31^. In brief, the gene of EBOV NP (residues 36–351 and 36–450) was cloned into the pET-21d expression vector, and the gene of MARV NP (residues 19–370) was cloned into the pET-28a expression vector (Novogen, China). Both NP proteins carry a 6× His tag fused at the C terminus. *E. coli* strain BL21 (DE3) (BioMed, China) was transformed with the expression vector and induced with isopropyl β-D-1-thiogalactopyranoside (IPTG) at an optical density at 600 nm of 0.6. Following overnight expression at 16 °C, bacterial cultures were harvested and lysed in the buffer of 20 mM Tris-HCl (pH 8.5) and 200 mM NaCl using an ultra-high pressure cell disrupter (JNBIO, China). Cleared lysates were applied to a Ni-NTA column and eluted with lysis buffer containing 0.5–1 M imidazole. Proteins were subsequently purified on a Superdex-200 column (GE Healthcare) with a void volume of ~ 8 mL. The purified EBOV NP and MARV NP were all > 95% pure according to SDS-PAGE analysis.

### Preparation of Chinese licorice extracts

The licorice powder (10 g) was macerated in 200 mL 70% (v/v) methanol aqueous solution and extracted by ultrasonication for 1 h. The extracted solution was filtered and concentrated to dryness using a combination of rotary evaporation under reduced pressure and lyophilization. The resulting powder of licorice extracts was stored at 4 °C ready for use.

### Affinity MS and metabolomic analysis of NP incubation with licorice extracts

The licorice extract powder was completely dissolved with 95% DMSO to make a stock solution of 160 mg/mL. The stock was diluted with the EBOV NP incubation buffer (20 mM Tris-HCl, 200 mM NaCl, 5 mM DTT, pH 8.5) or the MARV NP incubation buffer (20 mM Hepes, 200 mM NaCl, pH 7.5) to a concentration of 3.2 mg/mL. Then NP protein at 40 μM was mixed with the diluted licorice extract solution at 1:1 (v/v) to a final volume of 50 μL. Incubation was kept at 16 °C for 30 min. The protein incubation solution was then filtered through an ultrafiltration unit (30 kDa) by centrifugation at 13000 g for 10 min at 4 °C. The retained solution on the membrane was washed twice with 200 mM ammonium acetate. The resulting solution of the protein complex was transferred to a new centrifugal tube, and bound compounds were dissociated with 90% methanol in deionized water. After centrifugation, compounds in the supernatant were obtained and evaporated by speed vacuum, reconstituted in 20% methanol, and analyzed by liquid chromatography coupled to high-resolution mass spectrometry (LC-HRMS). The protein-free control was prepared by using the buffer substitute for NP during incubation. All samples were prepared in four replicates and analyzed separately.

The compound mixture was analyzed on a Waters Synapt high-definition mass spectrometer (Milford, MA) equipped with a Waters Acquity Ultra-performance liquid chromatography (UPLC) system. UPLC separation was carried out on a Thermo Hypersil GOLD C18 column (2.1 mm × 100 mm, 3 μm) at a flow rate of 200 μL/min, with the mobile phases water/0.1% formic acid (A) and acetonitrile/0.1% formic acid (B). The LC method was as follows: 0–1 min, B at 2%; 1–3 min, B at 2–10%; 3–9 min, B at 10–35%; 9–12 min, B at 35–80%; then kept at 80% for 4 min and re-equilibrate for 4 min. The ESI ion source operated in the negative ion mode. Mass spectra were acquired within a mass range of 100–1000 m/z, with capillary voltage 2.6 kV, sample cone voltage 45 V, extraction cone voltage 4.7 V, desolvation temperature 350 °C, source temperature 100 °C, and desolvation gas flow 500 L/h. Internal calibration with leucine–enkephalin (LE) infusion via a LockSpray™ interface achieved mass accuracy within 10 ppm. A diluted sample of the crude extract stock in 20% MeOH at 1.6 mg/mL was injected every 4 runs to ensure stability of the analytical platform. MSMS spectra were acquired on selected compound precursors with collision energy set at 45–55 eV and other ion source conditions identical to MS full scans.

### Data processing for NP ligand identification

The raw LC-HRMS data was processed with XCMS software^41^ for peak alignment and peak intensity *(i.e.*, peak area) determination. Extracted ion chromatograms (EICs) were checked to review consistency of integration across samples, to analyze peak shapes, and to exclude background noise. Features from the NP incubation sample showing intensity over 25 (LOQ levels for most compounds in our LC-HRMS system) across four replicates were kept for multivariate analysis. The preprocessed data sets were used as input for SIMCA (version14, Umetrics, Sweden), and data analysis was preceded with the supervised Orthogonal Projection to Latent Structures-Discriminant analysis (OPLS-DA) models^27^,^28^,^42^. All data were log transformed and Pareto scaled to minimize heteroscedasticity and to adjust for fold differences between components. The model validity was verified using permutation tests and CV-ANOVA by SIMCA and also by checking the goodness of fit (R^2^X = 0.908, R^2^Y = 0.993, Q^2^ = 0.967 for the EBOV NP model; R^2^X = 0.906, R^2^Y = 0.989, Q^2^ = 0.885 for the MARV NP model). Contribution of each component to sample group separation was reflected by the Variable Influence on Projection (VIP) score in OPLS-DA. We also calculated the ratio of peak intensity from the protein incubation sample *vs* the control (S/N) for each component. Components that match the accurate mass to the TCM compound database for Chinese licorice (within 20 mDa mass tolerance) and show S/N ratios with RSD < 30% from four replicates are recognized as putative licorice constituents, and their data were used to make the VIP and S/N plots (Fig. 1C). Potential NP ligands from licorice extracts were further selected based on: 1) VIP score > 1.0^27^,^28^; 2) average S/N ratio > 2.0 (RSD < 30%)^19^,^20^. The TCM compound database was purchased from Neotrident Company (Beijing, China)^43^.

### Affinity MS analysis of NP incubation with pure compounds

In the licorice compound mixture screens, 14 commercially available standards were mixed at a final concentration of 10 μM for each compound, and incubated with either NP protein at 20 μM. In the individual compound binding assay, two standards 18β-glycyrrhetinic acid and licochalcone A were separately incubated with either NP while keeping the protein and the compound both at 10 μM. Incubation samples of a total volume of 50 μL were prepared in the corresponding NP buffer described earlier, and maintained at 16 °C for 30 min. Ultrafiltration and complex dissociation were then conducted, and the resulting compound mixture or the specific ligand was analyzed by LC-HRMS using the same instrument as described earlier. The LC method for licorice extract analysis was also applied for compound mixture screens. In specific ligand binding assays, a short LC gradient from 2% to 80% B over the first 4 min and then kept at 80% B for 2 min was applied. The protein-free control was prepared using the buffer substitute for NPs and underwent the same process as the protein incubation sample. Each incubation sample and the corresponding control was prepared and analyzed in triplicate.

EICs of specific compounds were extracted using MassLynx software (v4.1, Waters, UK) based on the accurate mass measurement with a tolerance of 0.01 Da. The ratio of peak intensity from the protein incubation sample *vs* the control sample (S/N) was calculated for each compound to assess binding specificity. In the individual ligand binding assay, we also estimated the fraction of a specific compound associated with the NP protein (BF%) and its affinity (K_d_) under a given incubation condition using the method reported previously^21^.

### SPR analysis

The SPR experiment was performed on a Biacore T200 optical biosensor (GE Healthcare). Series S sensor chips CM5, NHS, EDC, ethanolamine HCl as well as sampling vials and caps were all obtained from Biacore. HEPES buffer (pH 7.5) with 5% DMSO was used as running buffer. The NP protein (50 μg/mL) in 10 mM sodium acetate buffer (pH 5.5) was injected for immobilization. During each binding cycle, a compound solution (100 μM) was injected for 1 min at a flow rate of 30 mL/min and dissociation was monitored for 300 s. Data were collected with the biosensor instrument thermo stated to 25 °C. Raw data were further processed to remove systematic artifacts from nonspecific binding, signal drift, and bulk refractive index changes. Solvent correction and molecular weight adjustment were applied. All data processing was performed using the Biacore T200 Evaluation Software.

### Fluorescence-based thermal shift (FTS) assay

The FTS assay was performed with Protein Thermal Shift Dye Kit^TM^ according to the user protocol (Thermo Fisher, USA). Both the EBOV-NP or MARV-NP protein and the test compound were diluted in the corresponding incubation buffer. The NP protein at 10 μM was then mixed with each compound at 200 μM and the FTS dye (1X) in a total volume of 20 μL. All reactions were prepared in a 96-well plate which was then sealed, shaken, and centrifuged at 1000 rpm for 1 min at 4 °C. Thermal scanning (25 to 95 °C at 0.03 °C/s) was conducted on a real-time PCR machine (LightCycler**®**480, Roche) and fluorescence intensity was continuously monitored. Curve fitting, calculation of the protein melting temperature (Tm) and generation of data reports were carried out using LightCycler**®** 480 software (v1.5.0, Roche).

### SEC-MALS analysis

Approximately 50 μg of purified NP protein was used for SEC-MALS experiments. Each selected compound from licorice was mixed with either NP protein in 2 or 5-fold molar excess, and the samples were incubated overnight at 16 °C. Light scattering data were collected on a DAWN-HELEOS II detector (Wyatt Technologies, CA, USA) in line with a Superdex-200 column running in buffer of 20 mM Tris (pH 8.5), 200 mM NaCl, and 5 mM DTT. Raw data was analyzed using ASTRA 6.1 software (Wyatt Technologies, CA, USA) to determine the weight averaged molecular mass.

### *In silico* docking of EBOV NP-ligand interactions

The coordinates and structural factors of EBOV NP were derived from RCSB (PDB code 4Z9P). Protein-ligand interaction was analyzed using Discovery Studio 4.1 (Neotrident, Beijing, China). The EBOV NP structure was pre-processed by adding hydrogen atoms. During docking simulation, the optimal conformations for each ligand-protein binding state were retreated in spacious searching space. Subsequent analysis of the interaction between specific compounds and EBOV NP was conducted using AutoDock (version 4.2). Structural cartoons were prepared using PyMOL^44^. Sequence alignment was performed using ClustalW^45^ and graphed using ESPript 3.0^45^.

### RNA binding assay

EBOV NP 36–450 was purified on the Superdex-200 column equilibrated with a high-salt buffer (20 mM Tris-HCl, 1.5 M NaCl, 5 mM DTT, pH 8.5). The fraction of monomers was collected and diluted by seven-fold to a low-salt condition to obtain NP oligomers free of RNA. Removal of RNA from the NP oligomer was confirmed by A260/A280 ratio measurement (<0.65).

The fluorescence polarization experiment was established as previously described^46^. An 18-nucleotide RNA (CGGACACACAAAAAGAAA) corresponding to the 5′ positives sense EBOV leader sequence and labeled at its 5′ terminus with carboxyfluoscein was purchased from TaKaRa Corporation (Dalian, China). The EBOV NP 36–450 oligomer was mixed with the RNA probe diluted in the FP buffer (10 mM MgCl_2_, 20 mM Tris-HCl, 200 mM NaCl, 5 mM DTT, pH 8.5) at a final concentration of 200 nM. The NP protein was serially diluted for RNA binding curve analysis. In the GC7 competition experiment, the EBOV NP 36–450 monomer at 50 μM was first incubated with GC7 at different concentrations before the mixture was diluted by seven-fold to a low-salt condition and the RNA probe was added at 200 nM. A control was prepared without adding GC7 to the monomer solution. Fluorescence anisotropy was read on a Flex Station 3 (Molecular Devices). Reported anisotropy signals are the average and standard deviations of three replicate experiments. K_d_ and IC_50_ were determined using software GraphPad Prism 5.

## Additional Information

**How to cite this article**: Fu, X. *et al*. Novel Chemical Ligands to Ebola Virus and Marburg Virus Nucleoproteins Identified by Combining Affinity Mass Spectrometry and Metabolomics Approaches. *Sci. Rep.* **6**, 29680; doi: 10.1038/srep29680 (2016).

## Supplementary Material

Supplementary Information

Supplementary Table 1

## Figures and Tables

**Table 1:** Binding properties of specific compounds to NPs measured by affinity MS.

Compound	EBOV NP			MARV NP		
	S/N^a^	BF%^b^	K_d_ (μM)	S/N	BF%	K_d_ (μM)
GC7	2.7 ± 0.3	11.0% ± 1.1%	50.0 ± 7.3	2.3 ± 0.4	16.9% ± 4.1%	89.7 ± 15.3
GC10	1.2 ± 0.1	0.1% ± 0.0%	NB^c^	1.8 ± 0.1	0.9% ± 0.5%	NB
GC13	6.0 ± 1.3	0.3% ± 0.0%	>1000	11.0 ± 1.9	0.1% ± 0.1%	>1000

SDs are shown for all measurements from independent experiments in triplicate.

^a^The ratio of peak intensity from the protein incubation sample *vs* the control.

^b^The fraction of a compound associated with the NP protein in complexes.

^c^No binding of the compound with S/N < 2.

**Table 2:** Thermal stability of NPs incubated with specific compounds assessed by the thermal shift assay.

Compound	EBOV NP	MARV NP
	△Tm (°C)^a^	△Tm (°C)
GC7	−9.2 ± 0.4	−1.1 ± 0.1
GC13	−3.2 ± 0.3	0.7 ± 0.6
GC10	−0.8 ± 0.4	−0.2 ± 0.3
GC1	−0.5 ± 0.2	−0.2 ± 0.3
GC2	0.0 ± 0.0	0.2 ± 0.3
GC3	−0.6 ± 0.1	−0.4 ± 0.3
GC4	0.1 ± 0.2	0.2 ± 0.3
GC5	−0.2 ± 0.3	0.0 ± 0.5
GC6	0.1 ± 0.2	−0.2 ± 0.3

^a^Change of the melting temperature (Tm) of the NP protein incubated with a compound relative to the free protein. Negative values indicate decrease of Tm. Mean values and standard deviation from triplicate measurements are shown for each condition.

**Table 3:** Monomer and oligomer distribution of NPs incubated with specific compounds assessed by SEC-MALS.

Compound added^a^	EBOV NP			MARV NP		
	Monomer (%)	Oligomer(%) >1000 kDa	Oligomer(%) <1000 kDa	Monomer (%)	Tetramer (%)	Oligomer(%) >1000 kDa
Protein only	100			64.7	31.6	3.7
GC7 (1:2)		31.3	68.7	71.0	22.8	6.2
GC7 (1:5)		100		84.5	8.6	6.9
GC13 (1:5)	100			29.0	68.3	2.6
GC10 (1:5)	64.0	36.0		63.3	36.2	0.5
GC2 (1:5)	100			66.3	29.7	4.0

^a^Each compound was mixed with the NP protein in 2-fold or 5-fold molar excess.
