# Supplementary material for: Novel Chemical Ligands to Ebola Virus and Marburg Virus Nucleoproteins Identified by Combining Affinity Mass Spectrometry and Metabolomics Approaches
Source: Sci Rep. 2016 Jul 12;6:29680. doi: 10.1038/srep29680 (PMC4940736; doi:10.1038/srep29680)
Supplement: Supplementary Information [file srep29680-s1.pdf]

Supporting Information for Manuscript

Novel Chemical Ligands to Ebola Virus and Marburg Virus Nucleoproteins Identified by Combining Affinity Mass Spectrometry and Metabolomics Approaches

Xu Fu[1,2#], Zhihua Wang[2,3#], Lixin Li[2#], Shishang Dong[2#], Zhucui Li[4], Zhenzuo Jiang[5], Yuefei Wang[5*], Wenqing Shui[6*]

Table S2. SPR response of NP incubated with different compounds

Compound	EBOV NP<br>Corr response (RU)[a]	MARV NP<br>Corr response (RU)
GC7	128	35.8
GC13	43.8	29.2
GC10	8.8	5.2
GC1	3.1	2.0
GC2	2.0	1.1
GC3	2.9	2.8

[a]The corrected SPR response above 10 RU indicates positive binding.

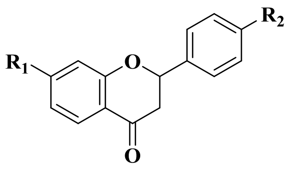

Compound No.	Compound name	R1	R2
GC2	Neoliquiritin	*O*-Glc	OH
GC12	Liquiritin	OH	*O*-Glc
GC1	Liquiritin apioside	OH	*O*-Glc-Api
GC8	Liquiritigenin	OH	OH

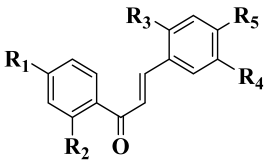

Compound No.	Compound name	R1	R2	R3	R4	R5
GC14	Isoliquiritin	OH	OH	H	H	*O*-Glc
GC4	Neoisoliquiritin	*O*-Glc	OH	H	H	OH
GC11	Isoliquiritigenin	OH	OH	H	H	OH
GC13	Licochalcone A	OH	H	OCH_3	$C(CH_3)_2CH=CH_2$	OH

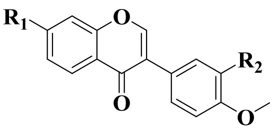

Compound No.	Compound name	R1	R2
GC5	Ononin	*O*-Glc	H
GC9	Calycosin	OH	OH

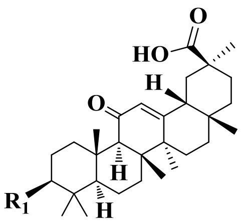

Compound No.	Compound name	R1
GC6	Glycyrrhizin	*O*-GlcA-GlcA
GC7	18*β*-Glycyrrhetinic acid	OH

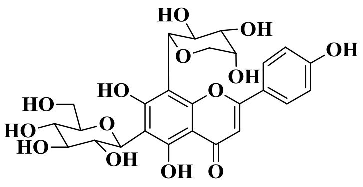

Compound No. GC3

Compound name: Schaftoside

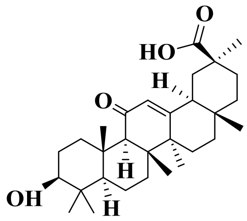

Compound No. GC10

Compound name:18*α*-Glycyrrhetinic acid

Figure S1. Names and chemical structures of compounds GC1-GC14 in Chinese licorice

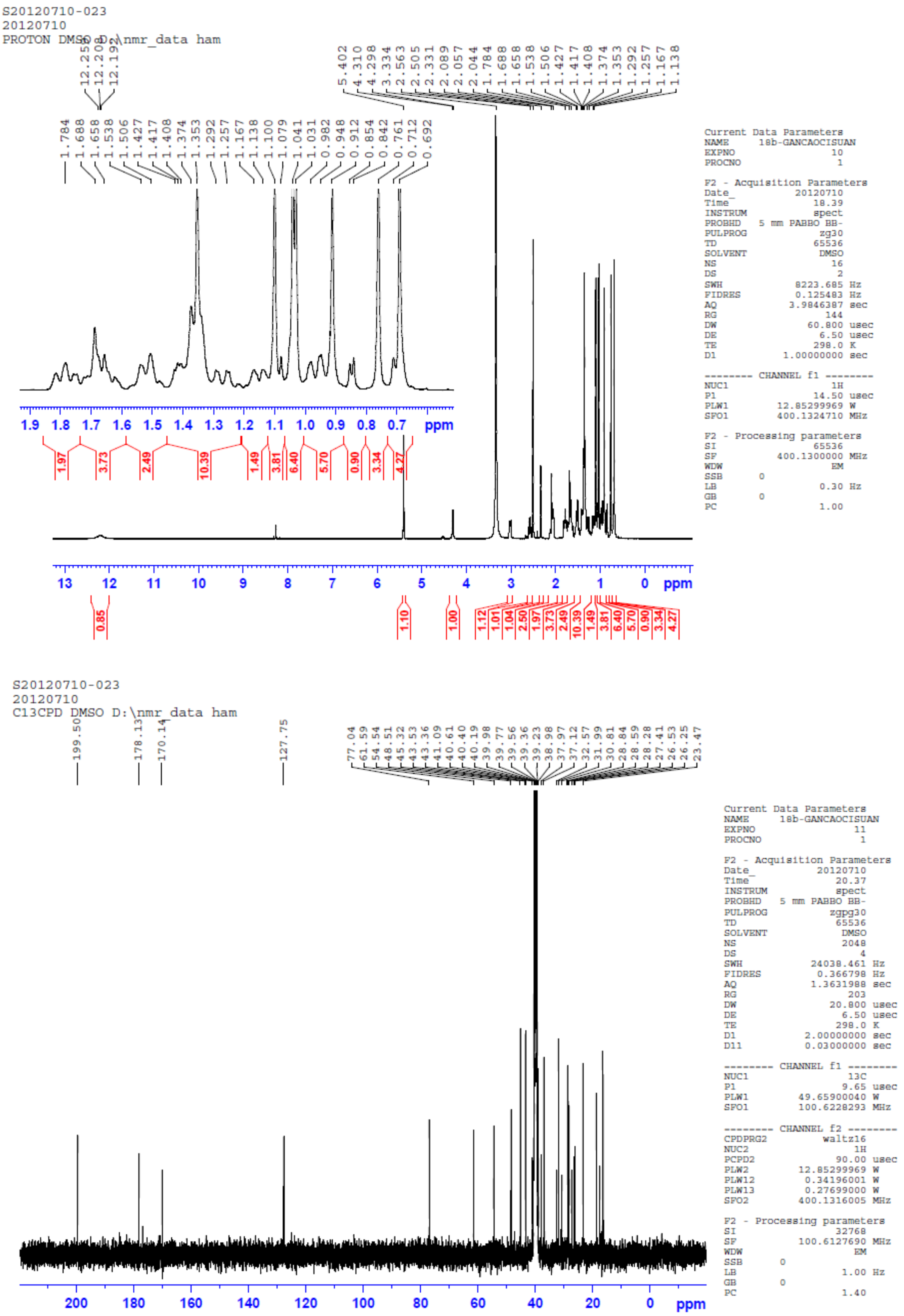

Figure S2-A. The upper ^{1}H-NMR spectrum (400 M, DMSO-d_6) and the lower ^{13}C-NMR spectrum (100 M, DMSO-d_6) for 18β-glycyrrhetinic acid (GC7)

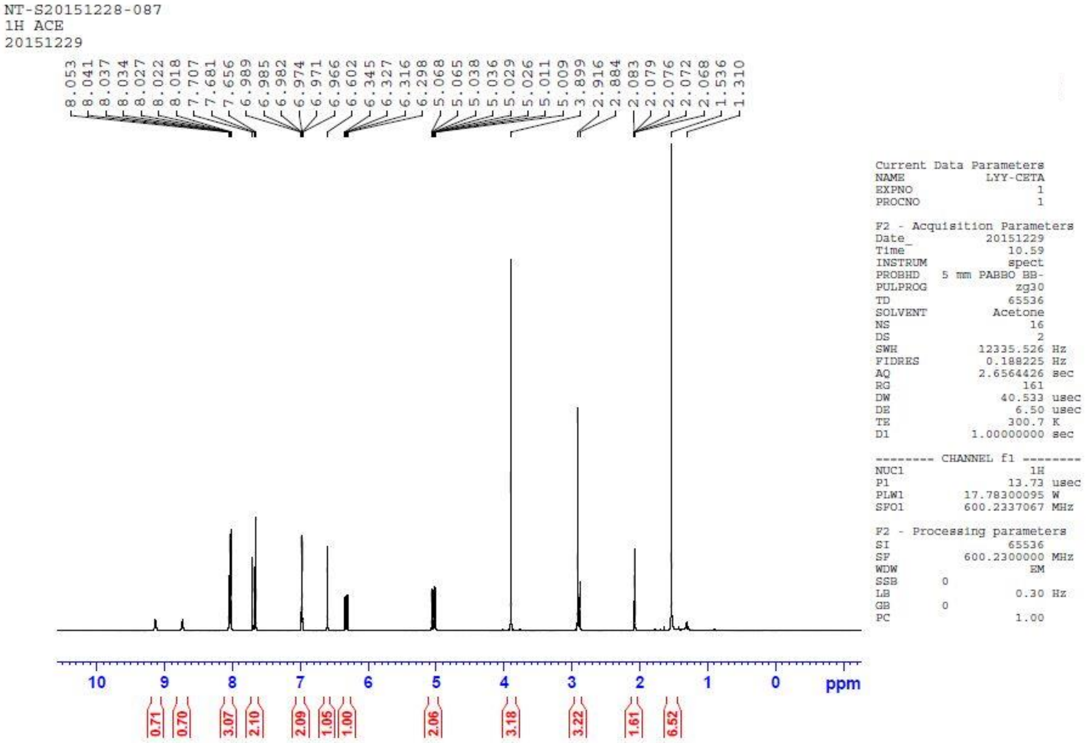

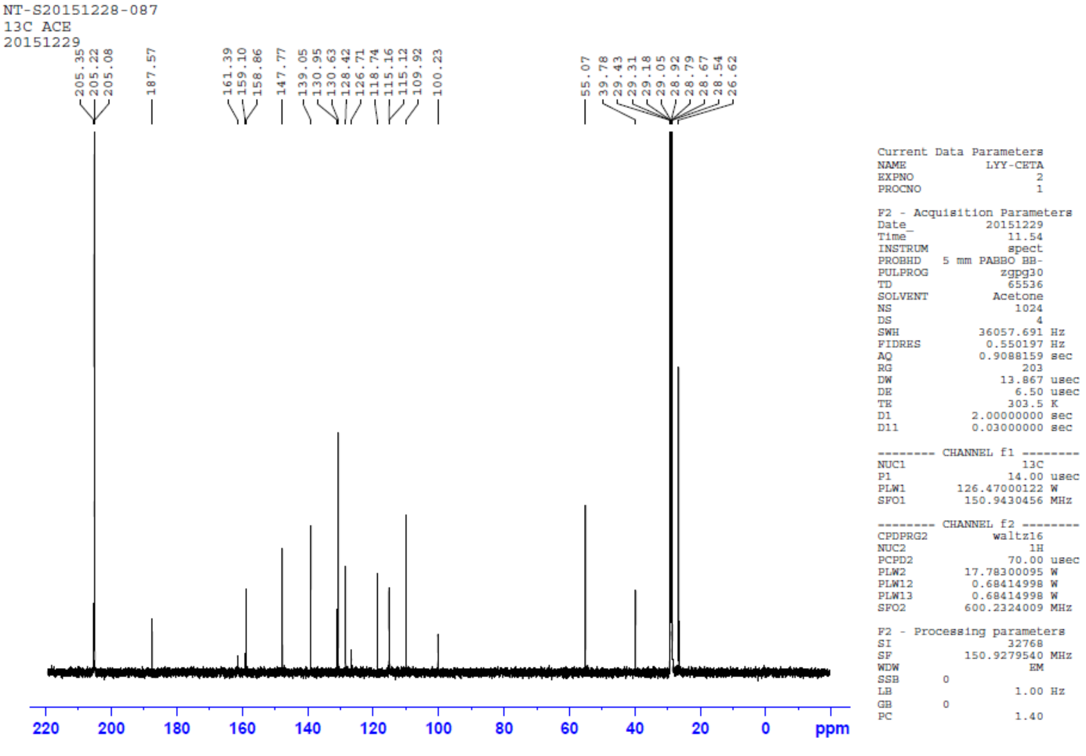

Figure S2-B. The upper ^{1}H-NMR spectrum (600 M, acetone-d_6) and the lower ^{13}C-NMR spectrum (150 M, acetone-d_6) for licochalcone A (GC13)

A

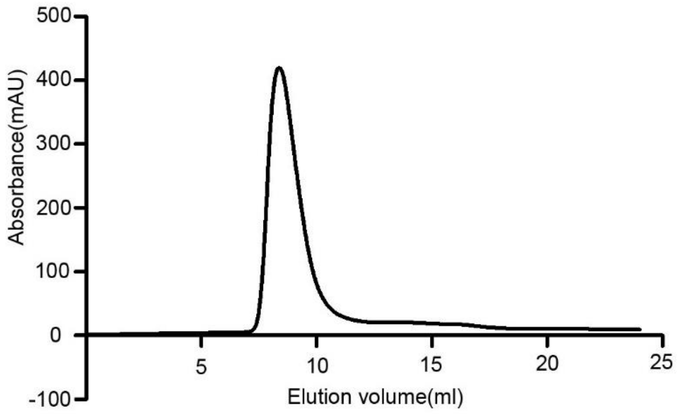

B

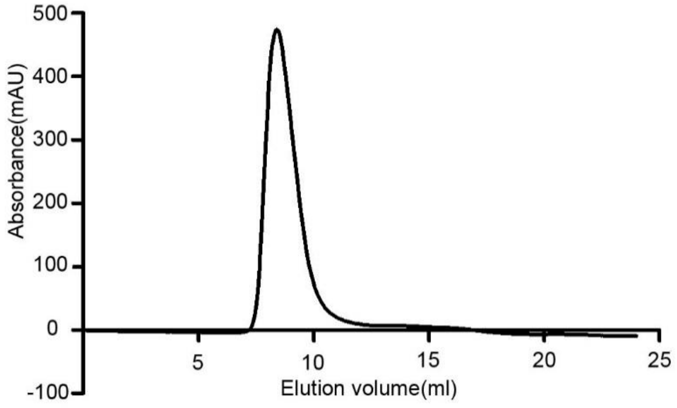

Figure S3. SEC chromatograms of EBOV NP_{core} 36-351 incubated with GC7 (A) and apo EBOV NP 1-450 known to form large homo-oligomeric complexes (B).

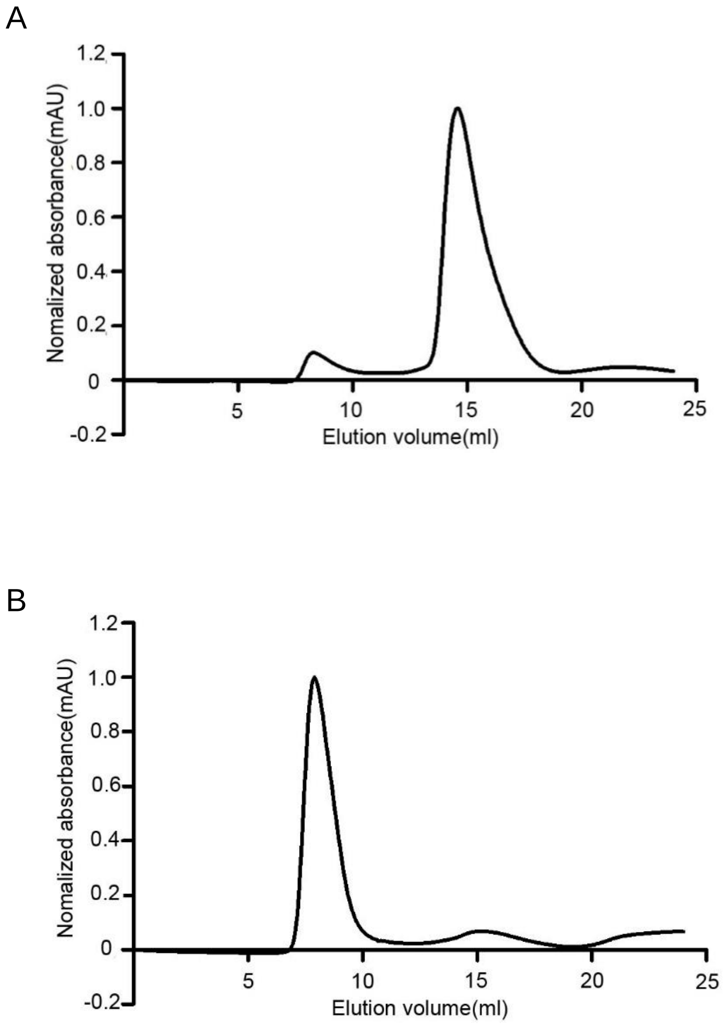

Figure S4. SEC chromatograms of EBOV NP 36-450 eluted in a high-salt buffer containing 1.5 M NaCl (A) and EBOV NP 36-450 diluted in a low-salt buffer containing 200 mM NaCl (B). The RNA-free oligomeric EBOV NP 36-450 purified in the low-salt buffer was used for the RNA binding assay.
